# A cluster-analytic approach towards multidimensional health-related behaviors in adolescents: the MoMo-Study

**DOI:** 10.1186/1471-2458-12-1128

**Published:** 2012-12-31

**Authors:** Sarah Spengler, Filip Mess, Nadine Mewes, Gert BM Mensink, Alexander Woll

**Affiliations:** 1 Sports Science, University of Konstanz, Universitätsstraße 10, Constance 78457, Germany; 2 Robert Koch-Institute, General Pape Straße 62-66, Berlin, 12101, Germany; 3 Karlsruhe Institute of Technology, Sports Science, Engler-Bunte-Ring 15, Karlsruhe, 76131, Germany

**Keywords:** Motorik-Modul (MoMo), Health-related lifestyle, Cluster analysis, German, Adolescents

## Abstract

**Background:**

Although knowledge on single health-related behaviors and their association with health parameters is available, research on multiple health-related behaviors is needed to understand the interactions among these behaviors. The aims of the study were (a) to identify typical health-related behavior patterns in German adolescents focusing on physical activity, media use and dietary behavior; (b) to describe the socio-demographic correlates of the identified clusters and (c) to study their association with overweight.

**Methods:**

Within the framework of the German Health Interview and Examination Survey for Children and Adolescents (KiGGS) and the “Motorik-Modul” (MoMo), 1,643 German adolescents (11–17 years) completed a questionnaire assessing the amount and type of weekly physical activity in sports clubs and during leisure time, weekly use of television, computer and console games and the frequency and amount of food consumption. From this data the three indices ‘physical activity’, ‘media use’ and ‘healthy nutrition’ were derived and included in a cluster analysis conducted with Ward’s Method and K-means analysis. Chi-square tests were performed to identify socio-demographic correlates of the clusters as well as their association with overweight.

**Results:**

Four stable clusters representing typical health-related behavior patterns were identified: Cluster 1 (16.2%)—high scores in physical activity index and average scores in media use index and healthy nutrition index; cluster 2 (34.6%)—high healthy nutrition score and below average scores in the other two indices; cluster 3 (18.4%)—low physical activity score, low healthy nutrition score and very high media use score; cluster 4 (30.5%)—below average scores on all three indices. Boys were overrepresented in the clusters 1 and 3, and the relative number of adolescents with low socio-economic status as well as overweight was significantly higher than average in cluster 3.

**Conclusions:**

Meaningful and stable clusters of health-related behavior were identified. These results confirm findings of another youth study hence supporting the assumption that these clusters represent typical behavior patterns of adolescents. These results are particularly relevant for the characterization of target groups for primary prevention of lifestyle diseases.

## Background

Adolescence is a critical period in life for adopting health behaviors, and these adopted health behaviors most probably track into adulthood [1-4]. Hence, understanding health behavior in adolescents is very important. It is well known that many adolescents in Germany do not meet the current physical activity recommendations, spend much time using electronic media, and eat too much processed meat and sweets and not enough vegetables and fruit compared to current recommendations [5-7]. Activity level and dietary habits have been recognized as key aspects of lifestyle that influence the risk for chronic diseases such as cardiovascular disease, diabetes, obesity, cancer and depression [8-10]. Hence, promoting a healthy lifestyle systematically, especially during adolescence is critical. However, primary prevention programs can only be implemented effectively if the specific characteristics of the target group are known. For instance, Carr stated that “there is a need for clearer definitions of target groups, their characteristics and particular needs” [11]. Because previous studies mainly focused on each one of the different health-related behaviors separately [12], currently little is known about the co-occurrence of these health-related behaviors.

The approach of clustering of health-related behaviors is based on the concept of health-related lifestyles [13] which originates from the work of Max Weber (1922) [14]. Health-related lifestyles comprise a person’s health-related behaviors, health-related attitudes and their socio-structural context. Cockerham [15] defined “health lifestyles” as “collective patterns of health-related behavior based on choices from options available to people according to their life chances”. Because it is assumed that a person’s health-related lifestyle is a composition of individual choices and social conditions [15], it is important to consider not only behavior patterns but also their socio-demographic correlates. This approach can be used to identify and precisely describe clusters of different behavior patterns.

To date, only few studies aimed to identify typical health-related behavior patterns and their association with socio-structural variables in adolescents [12,16-24]. While most studies identified three to seven clusters, these studies included different health-related behaviors in their analyses (e.g. tobacco use, dental care, alcohol consumption, playing sports with parents, sleep duration, doing homework), and hence the results of these studies are not comparable. Some of these studies focused on energy balance-related behaviors (amongst others) [12,16,20-24] and partly examined the association with overweight. Further, Ottevaere et al. [12] focused on physical activity, sedentariness and dietary behavior in European adolescents and identified five clusters representing typical behavior patterns with different overweight prevalence. Moreover, to date limited information is available on health-related behavior patterns in German adolescents’. All previous German studies were based on regionally restricted samples [25-27].

The aims of the study were (a) to identify typical health-related behavior patterns in German adolescents focusing on physical activity, media use and dietary behavior; (b) to describe the socio-demographic correlates of the identified clusters and (c) to study their association with overweight.

## Methods

### Data collection

Data was collected between 2003 and 2006 as part of the German Health Interview and Examination Survey for Children and Adolescents (KiGGS) [28] and the substudy ‘Motorik-Modul’ (MoMo) [29]. KiGGS was approved by the Federal Office for Data Protection and by the ethics committee of Charité University Medicine. Each parent and participant gave informed written consent before enrolment into the survey. The survey was conducted in accordance with the Declaration of Helsinki. The KiGGS study was conducted by the Robert Koch-Institute (RKI) in Berlin and represents a nationwide cross-sectional survey on the health status of children and adolescents from 0 to 17 years of age [28]. For the representative subsample of MoMo, comprehensive data on the physical fitness and physical activity of 4,529 children and adolescents aged between 4 and 17 years is available. Participants were recruited from the KiGGS population allowing for the inclusion of data obtained in the KiGGS survey. A detailed description of the sample, materials and methods of the MoMo-Study can be found in Woll et al. [29]. For the current study, a subsample of adolescents between 11 and 17 years was used.

### Measurements

#### Food consumption

In the KiGGS Survey, data on dietary intake was collected with a semi-quantitative food frequency questionnaire (FFQ) [30] covering 54 food items, of which 45 considered the frequency and amount of consumption of specific food groups. The FFQ was designed using experiences from the construction of the US National Institutes of Health diet history questionnaire [31]. The instrument was validated against a modified diet history instrument (DISHES) [32] and showed fair to moderate ranking validity (Spearman correlation coefficients from 0.22 to 0.69, most values 0.5 or higher), which is comparable to that of FFQs in the current literature [33]. Based on the FFQ data, a healthy nutrition score (HuSKY) [34] was developed comparing adolescents’ dietary behavior with current recommendations for adolescents [35,36]. The score ranges from 0 to 100 and reflects the overall diet quality, where a score of 100 represents that the recommendations were fully met. The development of the FFQ as well as the HuSKY has been previously described in detail [30,34].

#### Physical activity

Physical activity levels in adolescents were assessed using the MoMo physical activity questionnaire (MoMo-PAQ). Questions of the MoMo-PAQ had sufficient reliability (between k = 0.54 and k = 0.81, mean k = 0.66 (SD = 0.19) on item level) and validity (significant correlation between allover activity index and accelerometer Actigraph GT1X (Actigraph LLC, Pensacola, FL, USA) r = 0.29). These reliability and validity results were similar to those of other questionnaires for measuring physical activity in adolescents [37]. Participants were asked about the amount and type of their weekly physical activity in sports clubs and during their leisure time outside of sports clubs. The questionnaire included questions on the frequency (how many times per week), duration (in minutes) and type (which sport) of their physical activity. Participants could report data for at most four different sports in sports clubs as well as during leisure time. An activity index was defined including physical activity in sports clubs and that during leisure time outside of sports clubs. Each reported sport was coded with the expended energy as metabolic equivalent of task (MET) per hour [38] for computing intensity of the activity. For every reported sport in sports club and those during leisure time, a subindex (#PA*duration*MET/60) was calculated where #PA represented the number of times each week when this sport was performed in different settings, duration represented the number of minutes spent on each activity and MET corresponded to the estimated MET of the sport. The eight sport subindices were added to an overall activity index.

#### Electronic media use

In the KiGGS questionnaire, participants were asked about the daily amount of time they spent on watching TV, playing console games and using the computer. Answer categories were coded according to Lampert et al. [6] with the following values: ‘never’ = 0, ‘ca. 30 minutes’ = 0.5, ‘one to two hours’ = 1.5, ‘three to four hours’ = 3.5, ‘more than four hours’ = 5. A sum score of the three variables was calculated representing the daily amount using these electronic media [6].

#### Socio-economic status (SES)

Based on parental information on education, professional status, and household net income, the adolescents were classified into low, intermediate, or upper socioeconomic status (SES) [39]. Scores were computed separately for each parent, and the adolescent’s status was defined according to the higher parent’s score.

#### Migration background

Adolescents were coded as having a migration background if the adolescent immigrated to Germany and at least one parent was not born in Germany or if both parents immigrated or had no German citizenship. If the adolescent was raised by only one parent, the status of the single parent was considered.

#### Anthropometric measurements

Height was measured to the nearest 0.1 cm with a portable telescopic height measuring scale (Holtain Ltd., UK) with the adolescents standing upright without shoes. Body weight was measured to the nearest 0.1 kg with an electronic scale (Typ SECA) while participants where only wearing underwear. BMI was calculated as body weight divided by height squared (kg/m^2^). Age- and gender-specific cut points [40] were used to define adolescents as normal weight or overweight. In this study, the term overweight includes overweight as well as obese adolescents.

### Participants

Overall, 1,828 adolescents participated in MoMo. 21 participants were excluded because the information about the expended MET for the type of sport they performed was missing. Of the remaining 1,807 adolescents only those were included who had no missing data for physical activity in sports clubs and during leisure time as well as for time spent on watching television or playing computer or console games. Further, only those adolescents were included who reached the inclusion criteria for the HuSKY [34]. Finally, data for 1,643 adolescents were used for further analyses. This study population consisted of 832 males and 811 females (50.6 and 49.4%, respectively) with a mean age of 13.7 (± 1.9) years. 783 participants were aged 11 to 13 years, and 860 were aged 14 to 17 years (52.3%). 25.3% had a low SES, 50.6% had a medium SES and 23.9% had a high SES. 9.7% of the adolescents had a migration background. This subsample did not differ significantly from the total sample (considering both included and excluded cases) in terms of the socio-demographic items age, sex, SES and migration background.

### Statistical analysis

All statistical tests were performed in SPSS statistical software for Windows (release 20.0; SPSS Inc., Chicago, IL, USA). Cluster analysis was used because a comparison of different analytical methods showed that the use of cluster analysis provides “a rich understanding of behavioral patterns and the related demographic characteristics” [41]. Following the recommendations of Punj and Stewart [42], a combination of hierarchical and non-hierarchical cluster analysis was used to identify clusters with similar habits in physical activity, media use and diet. Data on these three variables was standardized with z-scores before clustering. An advance single-linkage cluster analysis was calculated to identify outliers. This procedure tends to build small clusters, while indicating outliers [43], and two cases were eliminated based on the results of the calculation. Subsequently, Ward’s method was used as a hierarchical cluster analysis based on squared Euclidean distances. This analysis allows identifying and comparing several possible cluster solutions. The best solution was identified using the Elbow method [44]. This solution was used as the starting partition for the next step, the non-hierarchical k-means cluster analysis. K-means analysis was used to further fine-tune the preliminary solution by optimizing the classification. Reliability and stability of the final cluster solution was tested by randomly taking a subsample (50%) of the total sample and repeating the analyses on this subsample. A Kappa degree of the cluster solution of the subsample with that of the total sample was calculated. Kappa = .90 indicated excellent agreement. Homogeneity of the final solution was given because the variance within each cluster was smaller than that between the clusters. ANOVA and post hoc Scheffé tests were used to reveal differences in terms of the three included indices between each cluster. Chi-square tests were used to identify differences between the clusters on the socio-demographic factors sex, age, SES, migration background as well as overweight. The significance level for all statistical tests was set a priori to *α* = .05 and adjusted using Bonferroni correction for the multiple Chi-square tests.

## Results

**Figure 1:**
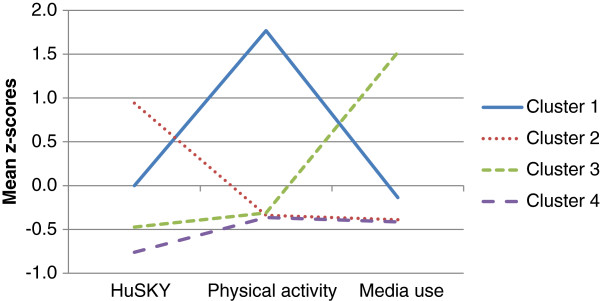
**Mean z-scores on the health behavior indices of the final cluster solution. **Cluster 1: high physical activity index, average media use and healthy nutrition indices; cluster 2: high healthy nutrition index, below average physical activity and media use indices; cluster 3: very high media use index, low physical activity and healthy nutrition indices; cluster 4: below average on all three indices.

The analyses revealed four stable clusters (Figure 1). The means of each cluster solution reported in z-scores as well as in row values are reported in Table 1.

**Table 1:** Mean values (z-scores) of the cluster solution, results of ANOVA

	**Cluster 1**	**Cluster 2**	**Cluster 3**	**Cluster 4**	**F**
N (%)	266 (16.2)	564 (34.3)	306 (18.6)	507 (30.9)	
HuSKY	0	0.94	−0.47	−0.76	605.68**
Mean ± SD	53.24 **±** 8.33	63.1 **±** 5.87	48.72 **±** 8.63	45.79 **±** 6.27	
Physical activity	1.77	−0.34	−0.31	−0.36	833.81**
Mean ± SD (MET/week)	71.11 **±** 23.55	16.18 **±** 13.69	16.89 **±** 17.54	15.51 **±** 13.79	
Media use	−0.14	−0.39	1.52	−0.41	643.81**
Mean ± SD (h/day)	2.85 **±** 1.75	2.29 **±** 1.30	6.56 **±** 2.14	2.24 **±** 1.08	

**p < .001.

*HuSKY*, healthy nutrition score.

Adolescents in cluster 1 had a high physical activity level. Post hoc Scheffé tests showed that the physical activity level of adolescents in cluster 1 (71.11 MET/week) differed significantly (p < .001) from that of adolescents in the other three clusters whose physical activity level was below average (below 17 MET/week in clusters 2, 3 and 4). Cluster 2 was characterized by a high healthy nutrition score (63.1). In contrast, adolescents in cluster 1 had at least an average healthy nutrition score (53.24) and healthy nutrition scores of adolescents in clusters 3 and 4 were below average (48.72 and 45.79 respectively). The differences in healthy nutrition score between all clusters were statistically significant (p < .001). While the media use index was high in cluster 3 (6.56 hours/day), values for this index were below average for clusters 1, 2 and 4 (2.85, 2.29 and 2.24 hours/day respectively). These differences were statistically significant between all clusters (p < .001) except between clusters 2 and 4 (p = .947). Overall, cluster 4 differs from all other clusters with below average scores on all three indices and the significantly lowest healthy nutrition score.

The socio-demographic correlates of the clusters are presented in Table 2. Chi-squares tests revealed that all correlates except ‘migration background’ differed significantly between clusters. In cluster 1 which was characterized by a high physical activity level, boys and adolescents with a high SES were overrepresented. Cluster 2 which had a high healthy nutrition score included more girls than boys and adolescents with a high SES were overrepresented. In the cluster with the highest index on media use (cluster 3), male adolescents, adolescents with a low SES and those with migration background were overrepresented. Cluster 4 (where all three indices were below average) included more girls than boys, and the distribution of age group and SES was similar to that of the total sample.

**Table 2:** Percentage of adolescents in each cluster for socio-demographic factors and overweight

		**Cluster 1**	**Cluster 2**	**Cluster 3**	**Cluster 4**	**Total**	**Chi Square**
Sex (adj. resid.)	**Male**	71.8 (7.5)	38.7 (−7.0)	69.3 (7.2)	41.6 (−4.9)	50.6	139.14**
	**Female**	28.2 (−7.5)	61.3 (7.0)	30.7 (−7.2)	58.4 (4.9)	49.4	
Age category (adj. resid.)	**11-13 yrs**	48.5 (0.3)	52.7 (2.9)	37.3 (−4.0)	47.9 (0.1)	47.7	19.02**
	**14-17 yrs**	51.5 (−0.3)	47.3 (−2.9)	62.7 (4.0)	52.1 (−0.1)	52.3	
SES (adj. resid.)	**Low**	18.6 (−2.8)	22.5 (−2.1)	35.5 (4.4)	26.5 (0.6)	25.5	39.99**
	**Medium**	54.0 (1.2)	48.7 (−1.1)	50.5 (0.0)	50.9 (0.2)	50.6	
	**High**	27.4 (1.4)	28.9 (3.4)	14.0 (−4.5)	22.6 (−0.9)	23.9	
Migration background (adj. resid.)	**Yes**	7.9 (−1.1)	8.3 (−1.4)	13.1 (2.2)	10.3 (0.5)	9.7	6.32
Overweight (adj. resid.)	**Yes**	12.5 (−1.6)	16.7 (0.7)	22.2 (3.4)	12.6 (−2.4)	15.8	15.85**

**p < .001.

*SES*, socio-economic status.

*adj. resid*, adjusted residual.

Chi Square tests revealed significant differences in the prevalence of overweight between the clusters (Table 2). Cluster 1 had the lowest relative number of overweight adolescents, and cluster 3 had the highest relative number of overweight adolescents. The percentage of overweight adolescents differed between cluster 3 (22.2%) and clusters 1 (12.5%; p = .002) and 4 (12.6%; p < .001), and that of cluster 2 did not differ significantly from the other three clusters.

## Discussion

### Identified clusters

In this study, four clusters representing typical health-related behavior patterns were identified. Cluster 1 can be characterized as the cluster with the most favorable health-related behavior pattern with a high physical activity level but ‘just’ average healthy nutrition scores and, ‘just’ moderate media use. These results showed that none of the typical behavior patterns identified in this study included most favorable behaviors for all three indices (high physical activity level, high healthy nutrition score, small amount of media use) but only for at most two indices: adolescents in cluster 2 showed favorable behaviors for nutrition and media use but not for physical activity level; adolescents in cluster 4 only show a favorable behavior for media use. Hence, as suggested by Ottevaere et al., adolescents appear to—consciously or unconsciously—compensate an unhealthy behavior in one dimension with healthy behavior in another dimension [12]. Only in cluster 3, the behaviors for all three indices can be seen as unfavorable suggesting low health awareness in adolescents in this cluster. Adolescents in cluster 4 are neither engaged in media consumption nor in physical activity and may prefer other leisure activities (e.g. playing music). In addition, the results of this study did not indicate that a high physical activity level excludes a high amount of media use. The characteristics of cluster 1 (high physical activity level and second highest media use index of all clusters) and cluster 4 (below average physical activity level and below average media use index) support the suggestion proposed by van der Horst et al. [45] and Biddle et al. [46] that a systematic association between physical activity level and media use is not compulsive. However, cluster 3 showed a very high media use index in combination with a very low physical activity level. Our data showed that in case of excessive media use a high physical activity level is not usual. It seems that adolescents who prioritize media use are usually not that strongly interested in other leisure activities.

Ottevaere et al. [12] studied adolescents of eight European cities and reported similar clusters of the three health indices. Although indices were generated using different methods, they also represented (i) the physical activity level (MVPA), (ii) sedentarism (media use) and (iii) diet quality [12]. In that study, five clusters were identified, and four of these clusters resembled the clusters we found in our study. The cluster that was only identified by Ottevaere et al. was the “active, low diet quality cluster”. However, it is possible that adolescents tending to belong to such a category were allocated to cluster 1 (high physical activity level) in our study. In addition, there are similarities in the socio-demographic correlates of health-related behavior identified in these two studies. In both studies, while girls were overrepresented in the clusters with high scores on healthy nutrition, boys were overrepresented in the clusters with high physical activity levels. Further, in these two clusters (“healthy cluster” and “inactive, high diet quality cluster”), Ottevaere et al. found a higher percentage of adolescents with highly educated parents and our study revealed that adolescents with a high SES were overrepresented in clusters 1 (high physical activity level) and 2 (high healthy nutrition score). Hence, as previously suggested by Ottevaere et al. [12], the identified clusters appear to be representative clusters for all adolescents or at least for adolescents in several European countries.

### Socio-demographic correlates

The described socio-demographic correlates of the four clusters are in agreement with current findings of correlative studies that showed that boys are more engaged in physical activity than girls [45]. Further, Sabbe [47] found similar gender differences in a cluster analysis of a sample of 10 year old children. In addition, our result that males were overrepresented in cluster 3 is in agreement with the finding that boys spend more time watching television and playing computer and console games than girls [45]. Moreover, older adolescents (14–17 years) were overrepresented in cluster 3 which may be explained by the notion that for adolescents aged between 11 and 13 years daily screen time may be more likely limited by the parents than for older adolescents. The association of clustering with the adolescents’ SES was as expected because previous studies reported that healthy nutrition was associated with a high SES [48] and high amounts of media use were associated with lower SES [45].

### Prevalence of overweight

Interestingly, the prevalence of overweight differed between the four clusters of health-related behavior. The high relative number of overweight adolescents in cluster 3 supports the suggestion that a high amount of media use is linked to higher body fatness [49]. This finding is further confirmed by the fact that cluster 4 comprised adolescents with similar physical activity level and healthy nutrition index as those of cluster 3 but had a smaller relative number of overweight adolescents than cluster 3. In addition, van der Sluis et al. [21] found a significantly higher BMI in the ‘unhealthy’ cluster compared to that in the ‘healthy’ cluster (total: four clusters). The results of our study indicate that the identified health-related behavior patterns are associated with the prevalence of overweight. Cluster 2 had the highest HuSKY value and a higher relative number of overweight people than clusters 1 and 4. Although the differences between these clusters were not significant, a high healthy nutrition score may not necessarily be related to lower overweight prevalence, at least not in adolescents. This assumption is supported by results of a review on the evidence of the association of dietary intakes and childhood obesity which revealed no consistent association between these two factors [50]. Similarly, other studies [12,47] did not find significant differences in BMI between the identified clusters of health-related behavior. Further, a part of the explanation may be “that people, who consume higher amounts of food, tend to meet the recommendation for adequate intake more often than people, who eat less food. Therefore persons, who eat more, tend to have higher scores” [51]. Another possible explanation for the relatively high prevalence of overweight in cluster 2 is the fact that overweight adolescents are more often on a weight reduction diet than normal weight adolescents [52]. Dieting is usually related to a higher diet quality, which is reflected by a higher HuSKY in our study. This assumption is confirmed by the fact that girls are overrepresented in this cluster, and studies show that females are more frequently reporting being on diet [52,53].

### Limitations

The approach to health-related behavior patterns employed in this study covered the behavioral aspects and considered the socio-structural context which are elements of the concept of health-related lifestyles, as mentioned in the introduction [13]. While this approach allowed for identifying typical behavior patterns and to specify their characteristics, it did not include health-related attitudes, the third component of the concept. Therefore, the clusters identified in this study represent rather health-related behavior patterns than health-related lifestyles. Considering this aspect in future research would provide further details on health-related behavior patterns because it can be assumed that health-related attitudes act as intermediaries for health-related behavior patterns.

All indices used in this study were based on self-administered questionnaires. However, statements on physical activity level and media use can be affected by the difficulty to recall the duration of activities among young people and summarizing as well as rounding this information. In addition, statements on diet behavior can be effected by the difficulty to remember the frequency and amount of food intake and by the subjective rating of portion sizes. The choice of collecting data by questionnaire was predetermined by the size of the survey population. While the three generated indices do not allow the assessment of detailed aspects of the health-related behaviors, they were adequate for achieving the aim of this study to provide an overall estimate of health-related behavior patterns. Furthermore, because cases with missing data were excluded, these results cannot be readily generalized for German adolescents. Nonetheless, the results of this study form the basis for future studies on the impact of cluster membership on the development of objective and subjective health parameters to determine the risk potential of each cluster for the development of chronic diseases and obesity. Once the risk potentials of the identified clusters are established, German adolescents could be categorized according to the reported behavior patterns. Those at high risk for the development of chronic diseases could be identified and interventions aimed at the specific needs of homogenous target groups could be developed.

## Conclusions

In this study, we identified typical health-related behavior patterns in German adolescents. Similar patterns were found in another European youth study. Based on the agreement of our results with the literature, we conclude that the identified clusters represent specific behavior patterns that are typical for adolescents at least in several European countries. The information on health-related behavior patterns and their correlates gained in this study makes a considerable contribution for the precise characterization of target groups for primary prevention of lifestyle diseases.

## Competing interests

The authors declare that they have no competing interests.

## Authors’ contributions

SS was responsible for the overall conception and design of this manuscript, statistical analysis and interpretation of data. FM and NM revised the manuscript. GM designed the FFQ of the KiGGS-Study and co-developed the HuSKY and revised the manuscript. AW designed the MoMo-Study. All authors read and approved the final manuscript.

## Pre-publication history

The pre-publication history for this paper can be accessed here:

http://www.biomedcentral.com/1471-2458/12/1128/prepub
